# PMD: A Resource for Archiving and Analyzing Protein Microarray data

**DOI:** 10.1038/srep19956

**Published:** 2016-01-27

**Authors:** Zhaowei Xu, Likun Huang, Hainan Zhang, Yang Li, Shujuan Guo, Nan Wang, Shi-hua Wang, Ziqing Chen, Jingfang Wang, Sheng-ce Tao

**Affiliations:** 1 Key Laboratory of Systems Biomedicine, Ministry of Education, Shanghai Center for Systems Biomedicine, Shanghai Jiao Tong University, Shanghai 200240, China; 2 State Key Laboratory of Oncogenes and Related Genes, Shanghai 200240, China; 3 Bio-ID center, School of Biomedical Engineering, Shanghai Jiao Tong University, Shanghai 200240, China; 4 Key Laboratory of Ministry of Education for Genetics, Breeding and Multiple Utilization of Crops, Fujian Agriculture and Forestry University, Fuzhou, Fujian 350002, China; 5 School of Life Science, Fujian Agriculture and Forestry University, Fuzhou, Fujian 350002, China; 6 The California Institute for Quantitative Biosciences (QB3), University of California, Berkeley, CA 94720, USA

## Abstract

Protein microarray is a powerful technology for both basic research and clinical study. However, because there is no database specifically tailored for protein microarray, the majority of the valuable original protein microarray data is still not publically accessible. To address this issue, we constructed Protein Microarray Database (PMD), which is specifically designed for archiving and analyzing protein microarray data. In PMD, users can easily browse and search the entire database by experimental name, protein microarray type, and sample information. Additionally, PMD integrates several data analysis tools and provides an automated data analysis pipeline for users. With just one click, users can obtain a comprehensive analysis report for their protein microarray data. The report includes preliminary data analysis, such as data normalization, candidate identification, and an in-depth bioinformatics analysis of the candidates, which include functional annotation, pathway analysis, and protein-protein interaction network analysis. PMD is now freely available at www.proteinmicroarray.cn.

Protein microarrays are miniaturized, parallel and high-throughput analysis systems, usually formed by spotting down hundreds to thousands of different proteins at high-density on a glass slide^1^,^2^,^3^,^4^. As a key technology of proteomics, protein microarrays have already been applied in a wide range of biological studies, including investigations of protein-protein interactions, protein-phospholipid interactions, small molecule targeting protein identification, biomarker identification, and protein posttranslational modifications^5^,^6^,^7^,^8^. Thousands of features can be simultaneously evaluated in a single experiment using a variety of protein microarrays, e.g., antibody microarray^9^, lectin microarray^10^,^11^, and proteome microarray^1^. New applications utilizing protein microarrays and novel protein microarray technologies are emerging continuously^12^,^13^.

At the moment, there are many specific databases for the storage and sharing of DNA microarray data, such as Gene Expression Omnibus (GEO)^14^ and ArrayExpress^15^ that employ well-established standards, such as Minimum Information About a Microarray Experiment (MIAME)^16^, for efficient data management and classification. However, by contrast, there is presently no specifically designed database for archiving and sharing of protein microarray data and no tailored standards for data processing and analyzing. As such, both GEO and ArrayExpress databases have collected some protein microarray data. However, these two databases are specifically designed for DNA microarrays: the protein microarray data are “bushes” interspersed in a huge “jungle” of DNA microarray data. Although DNA microarray specific MIAME standards have been applied for protein microarrays in GEO, it is obviously not ideally suited. Since there is only a few types of DNA microarray, there are many different types of protein microarray, which have much diversified applications. As such, a classification scheme that can include a broader range of protein microarray data is urgently needed.

To make protein microarray data fully accessible for further exploration, we constructed the Protein Microarray Database (PMD), which is specifically designed for archiving and analysis of protein microarray data. Importantly, to help users who are not familiar with protein microarray technology and protein microarray data processing, several bioinformatics tools have been integrated into PMD for protein microarray data processing and analyzing. The latest important publications about the development and applications of protein microarray technology are also actively collected in PMD and freely available for all the users. We strongly believe that this database could be a valuable resource for the research community. With the addition of the bioinformatics tools and the latest publications, PMD could serve as a unique port for protein microarray technology.

## Results

### PMD web interface

**Figure 1:**
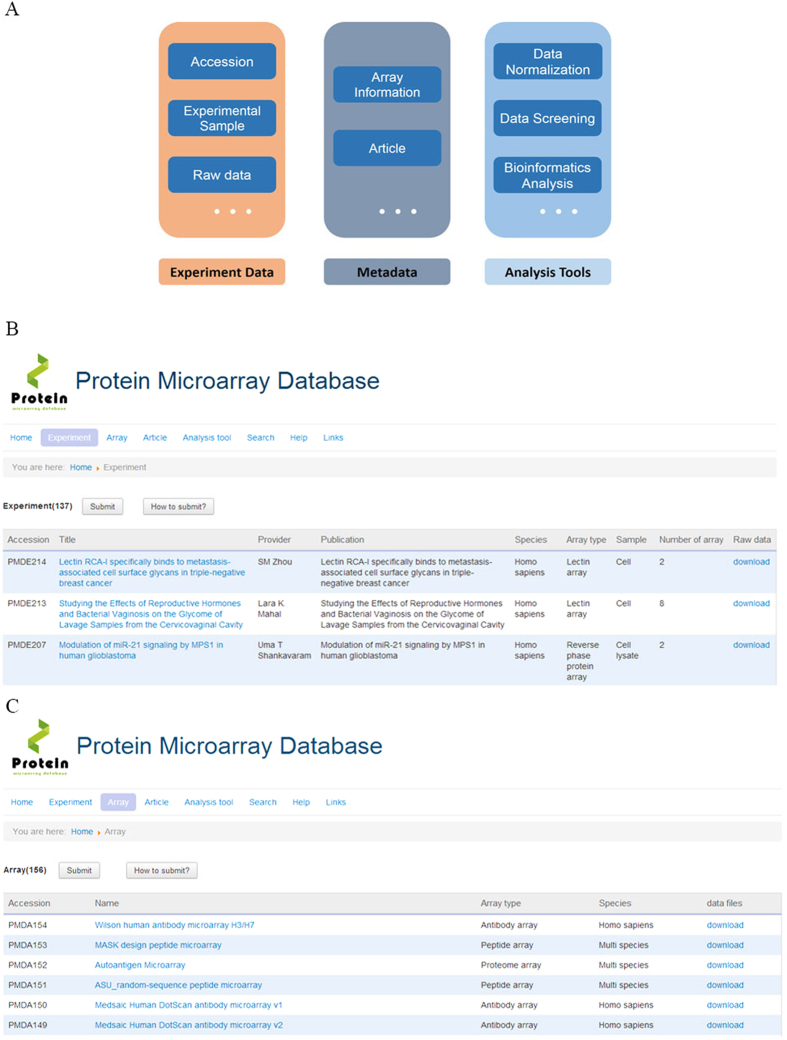
Overview of PMD. (**A**) PMD architecture. (**B**) Browsing the entire database by experiments. (**C**) Browsing the entire database by arrays.

The home page for PMD is a web-browser-based interface for performing database administration, data submission and storage, and query processing (Fig. 1A). Users can access the entire database by browsing the home page or submitting a query to search the database. To browse PMD, users can select the “Experiment” option or “Array” option in the home page, which will show the data based on the experiment names (or titles, as shown in Fig. 1B) and protein microarrays (Fig. 1C), respectively.

Besides, we are collecting protein microarray data from other databases, i.e. GEO & ArrayExpress and publications. Researchers who are developing their own protein microarrays or applying protein microarray for their own researches are highly encouraged to submit their original data to PMD. Following the archiving standards in PMD, users can submit their data by either microarray experiments (Fig. 1B) or microarrays (Fig. 1C). Since May 2014 when PMD began to accept data, there are now 137 experimental projects and 156 protein microarrays from 21 species, which could be classified into 7 microarray types, including proteome microarrays, antibody microarrays, lectin microarrays, etc.

### Analysis tools implemented in PMD database

PMD is not only a specific resource for archiving protein microarray data, but also a unique platform for integrated analysis. Like DNA microarrays, the raw data of protein microarrays are usually stored in two major formats: gpr file (GenePix) and txt file (Agilent). In PMD, we encouraged users to provide their raw data as gpr files. As raw data have to be processed before further data analysis, PMD provides a standard data processing and normalization protocol for new users. PMD adopts specifically designed R scripts for raw data normalization and identification of “differentially expressed proteins”. Here, “differentially expressed proteins” refers to proteins that show statistical differences between control microarrays and experimental microarrays. Additionally, PMD also provides bioinformatics tools for protein annotation and pathway analysis, which is achieved by combining The Database for Annotation, Visualization and Integrated Discovery (DAVID)^17^, Search Tool for the Retrieval of Interacting Genes/Proteins (STRING)^18^ and Protein ANalysis THrough Evolutionary Relationships (PANTHER)^19^. All of these analyses can be automatically performed after raw data were uploaded.

**Figure 2:**
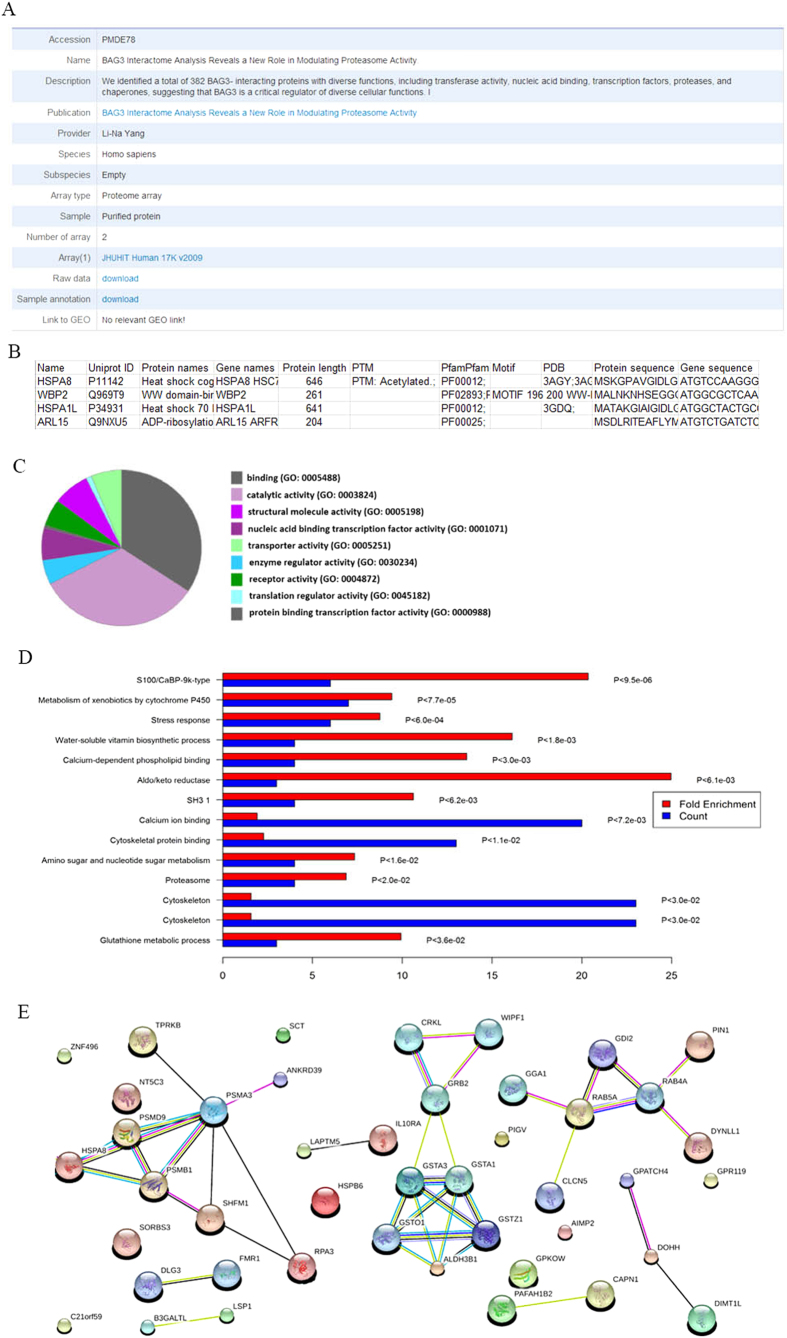
A case study for how to use the one-click analysis tools implemented in PMD. (**A**) Selected proteome microarray summary. (**B**) List of the “differentially expressed proteins”. (**C**) Molecular functions identified by PANTHER. (**D**) Pathway analysis for selected proteins using DAVID. (**E**) Protein-protein interaction network for selected proteins from STRING.

To clearly show how to use these analysis tools, we use a set of *Homo sapiens* proteome microarray data^20^ with PMD ID PMDE78 as an example (Fig. 2A). After submitting the data to PMD, and indicating the experimental and control groups, automatically, PMD will perform the analysis and generate the list of “differentially expressed proteins”. The list contains basic annotation, such as UniProt ID, Pfam information, Protein Data Bank (PDB) ID, and post-translational modification (Fig. 2B). One step further, PMD will automatically perform in-depth bioinformatics analysis based on the list of “differentially expressed proteins”. One can easily identify significantly enriched pathways by PANTHER (Fig. 2C), enriched gene ontology (GO) by DAVID (Fig. 2D), and protein-protein interaction (PPI) network by STRING (Fig. 2E). These results are included in a complete report, which will be automatically sent to the users.

## Discussion

**Figure 3:**
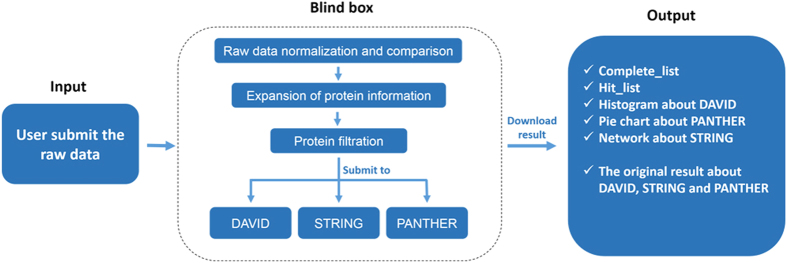
Work flow for PMD analysis tools. PMD analysis tools is an automated data analysis pipeline for protein microarray. After submitting protein microarray data into the database, by one-click PMD will automatically store the experimental and array information, normalize the raw data, and run the implemented analysis tools. In the end, users can receive a complete report containing a list of “differentially expressed proteins” and the results of all the in-depth bioinformatics analysis.

Compared to experiments using DNA microarrays, protein microarray experiments employ more diversified types of arrays and are designed to investigate a wider range of applications in both basic research and clinical studies. In this study, we report a specifically designed database for protein microarrays, named PMD. PMD has the following features: (I) It is a unique platform specifically designed for archiving original protein microarray data, and so it can promote data sharing among the proteomic community; (II) It provides standards and guidelines specifically tailored for the archiving and storage of protein microarray data; (III) Multiple software structures have been applied to construct an automated data analysis pipeline (Fig. 3). This pipeline is specific for protein microarrays, in contrast to the data analysis part of the GEO database that is more generally designed for DNA microarrays. In addition, the latest research publications about protein microarray technology development and application are also actively collected in PMD. With PMD, one can access all of the related information and the original protein microarray data in a “one-stop” fashion, with a capability of “one-click” data analysis. We strongly believe that PMD is a valuable resource for the research community by promoting protein microarray data sharing and facilitating data analysis.

## Methods

### Data acquisition and storage

The protein microarray data in PMD are obtained from 3 resources: the GEO/ArrayExpress databases, scientific literatures, as well as user’s contributions. PMD integrate GEO/ArrayExpress protein microarray data based on publications. Accordingly, several related datasets that are cited with a single publication are now stored as one experiment project in PMD. PMD also devote to collect protein microarray data that are associated with publications but are not publically available. In order to conveniently manage and share the protein microarray data, we implemented archiving standards for protein microarrays in PMD with specific modifications. These standards contain 6 critical elements: experiment name, provider, array type, sample type, microarray annotation, and raw data. Among these elements, array type and sample type are specifically designed for protein microarrays corresponding the diverse types and applications of protein microarrays.

### Database architecture and web interface

The collected protein microarray data were stored as a MySQL relational database. The information and raw data stored in PMD can be easily queried and downloaded by a user-friendly web interface. The front-end of PMD was constructed using Hypertext Preprocessor (PHP), while its back-end was built on joomla framework, running in an nginx web server. PMD architecture contains 3 major components: experimental management, metadata, and analysis tools.

## Additional Information

**How to cite this article**: Xu, Z. *et al.* PMD: A Resource for Archiving and Analyzing Protein Microarray data. *Sci. Rep.* **6**, 19956; doi: 10.1038/srep19956 (2016).
